# Biodegradability of Plastics

**DOI:** 10.3390/ijms10093722

**Published:** 2009-08-26

**Authors:** Yutaka Tokiwa, Buenaventurada P. Calabia, Charles U. Ugwu, Seiichi Aiba

**Affiliations:** 1 Okinawa Industrial Technology Center/12-2 Suzaki, Uruma, Okinawa 904-2234, Japan; E-Mail:ugutyaru@pref.okinawa.lg.jp (C.U.U.); 2 National Institute of Advanced Industrial Science and Technology (AIST)/Tsukuba Central 6, Higashi, Tsukuba Ibaraki 305- 8566, Japan; E-Mails:b.calabia@aist.go.jp (B.P.C.);aiba-seiichi@aist.go.jp (S.A.)

**Keywords:** aliphatic polyesters, bio-based plastics, biodegradability, enzymatic degradation, microbial degradation

## Abstract

Plastic is a broad name given to different polymers with high molecular weight, which can be degraded by various processes. However, considering their abundance in the environment and their specificity in attacking plastics, biodegradation of plastics by microorganisms and enzymes seems to be the most effective process. When plastics are used as substrates for microorganisms, evaluation of their biodegradability should not only be based on their chemical structure, but also on their physical properties (melting point, glass transition temperature, crystallinity, storage modulus etc.). In this review, microbial and enzymatic biodegradation of plastics and some factors that affect their biodegradability are discussed.

## 1. Introduction

With the advances in technology and the increase in the global population, plastic materials have found wide applications in every aspect of life and industries. However, most conventional plastics such as polyethylene, polypropylene, polystyrene, poly(vinyl chloride) and poly(ethylene terephthalate), are non biodegradable, and their increasing accumulation in the environment has been a threat to the planet. To overcome all these problems, some steps have been undertaken. The first strategy involved production of plastics with high degree of degradability.

**Figure 1.**
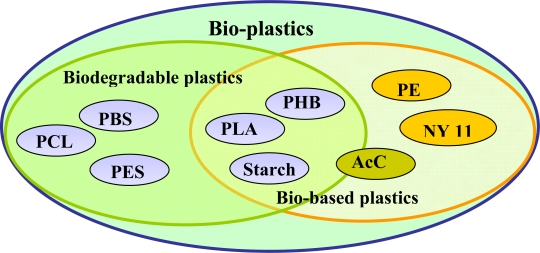
Bio-plastics comprised of biodegradable plastics and bio-based plastics.

The word ‘bio-plastic’ is used confusingly. In our understanding, however, bio-plastics consist of either biodegradable plastics (*i.e.,* plastics produced from fossil materials) or bio-based plastics (*i.e.,* plastics synthesized from biomass or renewable resources). The inter-relationship between biodegradable plastics and bio-based plastics is shown in Figure 1. Polycaprolactone (PCL), and poly(butylene succinate) (PBS) are petroleum based, but they can be degraded by microorganisms. On the other hand, poly(hydroxybutyrate) (PHB), poly(lactide) (PLA) and starch blends are produced from biomass or renewable resources, and are thus biodegradable. Despite the fact that polyethylene (PE) and Nylon 11 (NY11) can be produced from biomass or renewable resources, they are non-biodegradable. Acetyl cellulose (AcC) is either biodegradable or non-biodegradable, depending on the degree of acetylation. AcC’s with a low acetylation can be degraded, while those with high substitution ratios are non-biodegradable.

Biodegradable plastics are seen by many as a promising solution to this problem because they are environmentally-friendly. They can be derived from renewable feedstocks, thereby reducing greenhouse gas emissions. For instance, polyhydroxyalkanoates (PHA) and lactic acid (raw materials for PLA) can be produced by fermentative biotechnological processes using agricultural products and microorganisms [1–3]. Biodegradable plastics offer a lot of advantages such as increased soil fertility, low accumulation of bulky plastic materials in the environment (which invariably will minimize injuries to wild animals), and reduction in the cost of waste management. Furthermore, biodegradable plastics can be recycled to useful metabolites (monomers and oligomers) by microorganisms and enzymes. A second strategy involves degradation of some petroleum-derived plastics by biological processes. A typical example can be seen in the case of some aliphatic polyesters such as PCL and PBS that can be degraded with enzymes and microorganisms [4–6]. Studies have also shown that polycarbonates (particularly the aliphatic types) possess some degree of biodegradability [7].

Thirdly, bold attempts are being made to recycle non-biodegradable plastics. For instance, polystyrene (used in making some disposable spoons, plates, cups and some packaging materials) can be recycled and used as filler for other plastics.

Prior to the widespread applications of biodegradable plastics, it is important to evaluate and understand the mechanisms involved and the microorganisms that are associated with biodegradation. Regarding microbial and enzymatic degradation of plastics, we will discuss them from two sides: one aspect is based on microbial (enzyme) characteristics and the other is on characteristics of the plastics. Microbial (enzyme) characteristics imply distribution and kinds of microorganisms, as well as their growth conditions (such as, pH, temperature, moisture content, oxygen, nutrients, etc.), and types of enzymes (intracellular and extracellular enzyme, exo- or endo- cleavage types). An interesting question is, ‘what are the characteristics of plastics that can effectively promote the biodegradability of plastics?’ Usually our main focus has been on the chemical structure of polymers with respect to the biodegradability of water-soluble polymeric materials.

When the biodegradability of solid polymers is assessed, aside from their chemical properties, we should also note their physical properties as polymer aggregates. In other words, we should consider not only the first order structures but also the high-order structures of polymers that play an important role in the biodegradation process. Furthermore, it is worth mention that surface conditions (surface area, hydrophilic, hydrophobic properties) of plastics also generally influence the biodegradation mechanism of plastics. In this review, we will be discuss the biodegradation of plastics by both microbial and enzymatic processes and several factors that govern their biodegradability.

## 2. Biodiversity and Occurrence of Polymer-Degrading Microorganisms

Biodiversity and occurrence of polymer-degrading microorganisms vary depending on the environment, such as soil, sea, compost, activated sludge, etc. It is necessary to investigate the distribution and population of polymer-degrading microorganisms in various ecosystems. Generally, the adherence of microorganisms on the surface of plastics followed by the colonization of the exposed surface is the major mechanisms involved in the microbial degradation of plastics. The enzymatic degradation of plastics by hydrolysis is a two-step process: first, the enzyme binds to the polymer substrate then subsequently catalyzes a hydrolytic cleavage. Polymers are degraded into low molecular weight oligomers, dimers and monomers and finally mineralized to CO_2_ and H_2_O.

The clear zone method with agar plates is a widely used technique for screening polymer degraders and for assessment of the degradation potential of different microorganisms towards a polymer. Agar plates containing emulsified polymers are inoculated with microorganisms and the presence of polymer degrading microorganisms can be confirmed by the formation of clear halo zones around the colonies. This happens when the polymer-degrading microorganisms excrete extracellular enzymes which diffuse through the agar and degrade the polymer into water soluble materials. Using this technique, it was confirmed that PHB, polypropiolactone (PPL) and PCL degraders are widely distributed in different environments [8–10]. Majority of the strains that are able to degrade PHB belong to different taxa such as Gram-positive and Gram-negative bacteria, *Streptomyces* and fungi [9]. It has been reported that 39 bacterial strains of the classes *Firmicutes* and *Proteobacteria* can degrade PHB, PCL, and PBS, but not PLA [10]. Only a few PLA degrading microorganisms have been isolated and identified. The population of aliphatic polymer-degrading microorganisms in different ecosystems was found to be in the following order: PHB = PCL > PBS > PLA [8,11].

## 3. Factors Affecting the Biodegradability of Plastics

The properties of plastics are associated with their biodegradability. Both the chemical and physical properties of plastics influence the mechanism of biodegradation. The surface conditions (surface area, hydrophilic, and hydrophobic properties), the first order structures (chemical structure, molecular weight and molecular weight distribution) and the high order structures (glass transition temperature, melting temperature, modulus of elasticity, crystallinity and crystal structure) of polymers play important roles in the biodegradation processes.

**Figure 2.**
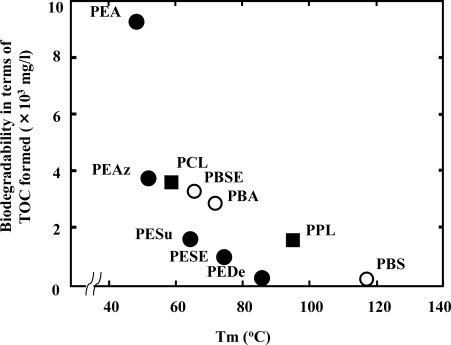
Relationship between Tm and biodegradability of polyesters by *R. arrhizus* lipase. PEA: poly(ethylene adipate); PESu: poly(ethylene suberate); PEAz: poly(ethylene azelate); PESE: poly(ethylene sebacate); PEDe: poly(ethylene decamethylate); PBS: poly(butylene succinate); PBA: poly(butylene adipate); PBSE: poly(butylene sebacate); PCL: polycaprolactone; PPL: polypropiolactone.

In general, polyesters with side chains are less assimilated than those without side chains [4]. The molecular weight is also important for the biodegradability because it determines many physical properties of the polymer. Increasing the molecular weight of the polymer decreased its degradability. PCL with higher molecular weight (Mn > 4,000) was degraded slowly by *Rhizopus delemar* lipase (endo-cleavage type) than that with low Mn [12]. Moreover, the morphology of polymers greatly affects their rates of biodegradation. The degree of crystallinity is a crucial factor affecting biodegradability, since enzymes mainly attack the amorphous domains of a polymer. The molecules in the amorphous region are loosely packed, and thus make it more susceptible to degradation. The crystalline part of the polymers is more resistant than the amorphous region. The rate of degradation of PLA decreases with an increase in crystallinity of the polymer [13,14]. As shown in Figure 2, the melting temperature (Tm) of polyesters has a strong effect on the enzymatic degradation of polymers. The higher the Tm, the lower the biodegradation of the polymer [12,15,16]. In general, Tm is represented by the following formula:

Tm=ΔH/ΔS

where ΔH was the change of enthalpy in melting and ΔS is the change of entropy in melting. It is well known that the interactions among polymer chains mainly affect the ΔH value and that the internal rotation energies corresponding to the rigidity (the flexibility) of the polymer molecule remarkably affect the ΔS value.

The chemical structures of aliphatic polyester, polycarbonate, polyurethane and polyamides, together with their (Tm)s are listed in Table 1. The aliphatic polyesters [ester bond (-CO-O-)] and polycarbonates [carbonate bond (-O-CO-O-)] are two typical plastic polymers that show high potential for use as biodegradable plastics, owing to their susceptibilities to lipolytic enzymes and microbial degradation. Compared with aliphatic polyesters and polycarbonates, aliphatic polyurethane and polyamides (nylon) have higher Tm values. The high (Tm)s of polyurethane and polyamide (nylon) are caused by the large ΔH value due to the presence of hydrogen bonds among polymer chains based on the urethane bond (-NH-CO-O-) and the amide bond (-NH-CO-) respectively.

On the contrary, the high Tm of aromatic polyester is caused by the small ΔS value with increase in the rigidity (decrease in flexibility) of the polymer molecule based on an aromatic ring.

## 4. Aliphatic Polyesters from Fossil Resources

### 4.1. Poly(Ethylene Adipate) (PEA)

PEA ([-OCH_2_CH_2_OOC(CH_2_)_4_CO-]n) is a pre-polymer of polyurethane. It is often blended with other polyesters to get specific desirable properties such as soft segments. PEA-degrading microorganisms were screened and isolated using PEA (Mn 3,000) as a sole source of carbon. Among the isolated PEA-degrading microorganisms, *Penicillium* sp. strain 14-3 exhibited the strongest activity. PEA was degraded in 120 h at high cell concentrations. This strain can degrade not only PEA but also aliphatic polyesters such as poly(ethylene succinate) (PES), PBS and poly(butylene adipate) (PBA) [17]. The enzyme responsible for the degradation of PEA has been purified and is considered to be a kind of lipase with broad substrate specificity. The purified enzyme has a molecular weight of 25 kDa and could degrade various kinds of aliphatic polyesters, such as poly(β-propiolactone) (PPL) and poly(ɛ-caprolactone) (PCL), but not poly(dl-3-methylpropiolactone) or poly (dl-3-hydroxybutyrate) [5]. This enzyme can also hydrolyze plant oils, triglycerides and methyl esters of fatty acids. Given that the purified enzyme of *Penicillium* sp. strain 14-3 has properties that are similar to lipase, some commercially available lipases and esterases were used to confirm if they were capable of degrading PEA. Results showed that lipases from *R. arrizus, R. delemar, Achromobacter* sp. and *Candida cylindracea* and esterase from hog liver showed activities on PEA and PCL [6].

### 4.2. Poly(ɛ-Caprolactone) (PCL)

PCL ([-OCH_2_CH_2_CH_2_CH_2_CH_2_CO-]n) is a biodegradable synthetic partially-crystalline polyester with low melting point (60 °C) and a glass transition temperature (Tg) of −60 °C. It is prepared by ring-opening polymerization of ɛ-caprolactone. PCL has been shown to be degraded by the action of aerobic and anaerobic microorganisms that are widely distributed in various ecosystems. Furthermore, the degradation of high molecular weight PCL was investigated using *Penicillium* sp. strain 26-1 (ATCC 36507) isolated from soil. PCL was almost completely degraded in 12 days. This strain can also assimilate unsaturated aliphatic and alicyclic polyesters but not aromatic polyesters [4]. A thermotolerant PCL-degrading microorganism which was identified as *Aspergillus* sp. strain ST-01 was isolated from soil. PCL was completely degraded by this strain after 6 days incubation at 50 °C [18]. PCL and PHB were degraded under anaerobic condition by new species of microorganisms belonging to the genius *Clostridium* [19].

PCL can be degraded by lipases and esterases [6]. The degradation rate of PCL is dependent on its molecular weight and degree of crystallinity. Enzymatic degradation of PCL by *Aspergillus flavus* and *Penicillium funiculosum* showed that faster degradation was observed in the amorphous region [20].

The biodegradability of PCL can be increased by copolymerization with aliphatic polyesters [21,22]. In general, copolymers have lower crystallinity and lower Tm than homopolymers, and are thus more susceptible to degradation.

The susceptibility of PCL films which were prepared at different quenching temperatures (−78, 0, 25, 50 °C) by *R. arrhizus* lipase was evaluated at 30 °C. Large spherulites were formed on PCL film quenched at 50 °C but no spherulites were observed on PCL film quenched at −78 °C. X-ray diffraction diagram of PCL films quenched at 25 °C and 50 °C indicated the growth of PCL crystal in the direction of c-axis (thickness of crystal unit), but crystallinity did not increase so much (about 40%) with the rise in quenching temperature. The susceptibility of PCL film quenched at −78 °C was the highest and the susceptibility decreased with increase in quenching temperature. It was confirmed that the size of spherulites is an important factor for biodegradation of PCL. In addition, the storage modulus of PCL film samples increased with increase in quenching temperature. PCL film quenched at 50 °C had the highest storage modulus, while low storage modulus was observed on PCL film quenched at −195 °C. Storage modulus of PCL film also increased with increase in draw ratio. Furthermore, the susceptibility of PCL films to *R. arrhizus* lipase decreased with increase in draw ratio. As the storage modulus of polyesters can be determined over a wide range (from below Tg up to Tm), we could predict the rate of enzymatic degradation of polyesters by using the value of storage modulus of polyesters at 30 °C (the typical evaluation temperature of biodegradability) [23].

### 4.3. Poly (β-Propiolactone) PPL

PPL ([-OCH_2_CH_2_CO-]n) is a chemosynthetic biodegradable aliphatic polyester with good mechanical properties. The structural units of this polyester are similar to PHB and PCL, thus, it can be degraded both by PHB depolymerase and lipase [6,24,25]. Many PPL-degrading microorganisms are widely distributed in various environments and majority of these microorganisms belong to *Bacillus* sp. [26]. PPL-degrading microorganisms were isolated from different ecosystems and out of 13 isolates, nine of these strains were identified as *Acidovorax* sp., *Variovorax paradoxus*, *Sphingomonas paucimobilis.* PHB was also degraded by these isolates [27]. *R. delemar* can also degrade PPL [6]. Moreover, a novel PHB depolymerase from a thermophilic *Streptomyces* sp. was also capable of degrading PPL [28].

### 4.4. Poly(Butylene Succinate) (PBS) and Poly(Ethylene Succinate) (PES)

PBS ([-O(CH_2_)_4_OOC(CH_2_)_2_CO-]n) and PES ([-O(CH_2_)_2_OOC(CH_2_)_2_CO-]n) are aliphatic synthetic polyesters with high melting points of 112–114 °C and 103–106 °C, respectively. They are synthesized from dicarboxylic acids (*e.g.,* succinic and adipic acid) and glycols (*e.g.,* ethylene glycol and 1,4-butanediol) [29]. Their mechanical properties are comparable to polypropylene and low-density polyethylene (LDPE).

PBS degrading microorganisms are widely distributed in the environment, but their ratio to the total microorganisms is lower than PCL-degraders. The degradation of PBS by *Amycolatopsis* sp. HT-6 was investigated and results showed that this strain can degrade not only PBS but also PHB and PCL [30]. Several thermophilic actinomycetes from Japan Culture of Microorganisms (JCM) were screened for their capability of degrading PBS. *Microbispora rosea*, *Excellospora japonica* and *E. viridilutea* formed clear zone on agar plates containing emulsified PBS. *M. rosea* was able to degrade 50% (w/v) of PBS film after eight days cultivation in liquid medium [31].

PES is another chemosynthetically aliphatic polyester which is prepared either by ring-opening polymerization of succinic anhydride with ethylene oxide or by polycondensation of succinic acid and ethylene glycol [32]. In contrast with microbial polyesters which are susceptible to degradation in various environments, the degradability of PES was found to be strongly dependent on environmental factors [33]. Moreover, PES-degrading microorganisms have limited distribution in the environment in comparison with PHB and PCL-degrading microorganisms. A thermophilic *Bacillus* sp. TT96, a PES degrader was isolated from soil. This bacterium can also form clear zones on PCL and PBS plates but not on PHB [34]. A number of mesophilic PES-degrading microorganisms were isolated from aquatic and soil environments. Phylogenetic analysis revealed that the isolates belong to the genera *Bacillus and Paenibacillus*. Among the isolates, strain KT102 which is related to *Bacillus pumilus* was chosen since it could degrade PES film at the fastest rate among the isolates. This strain can degrade PES, PCL and olive oil but not PBS, PHB and PLA [35]. In addition, several fungi were isolated from various ecosystems and the isolates formed clear zones around the colony on agar plates containing PES. A strain NKCM1003 belonging to *Aspergillus clavatus* was selected and it can degrade PES film at a rate of 21μg/cm^2^/h. [36]. Comparative studies on the biodegradability of three poly(alkylene succinate)s [PES, PBS and poly(propylene succinate) (PPS)] with the same molecular weight were investigated using *R. delemar lipase.* PPS with low Tm (43–52 °C) had the highest biodegradation rate followed by PES, owing to the lower crystallinity of PPS compared to PES and PBS [37].

### 4.5. Aliphatic-Aromatic Copolyesters (AAC)

Due to the limited properties of many biodegradable aliphatic types of polyester that are important for many applications, attempt was made to combine the biodegradability of aliphatic polyesters with the good material properties of aromatic polyesters.

It has been reported that AAC, which consisted of PCL and aromatic polyester such as poly(ethylene terephthalate) (PET), poly(butylene terephthalate) (PBT) and poly(ethylene isophthalate) (PEIP) was hydrolyzed by *R. delemar* lipase [15]. The susceptibility of these AAC’s to hydrolysis by *R. delemar* lipase decreased rapidly with increase in aromatic polyester content. The susceptibility to lipase of AAC (which consisted of PCL and PEIP, and the latter being used as a low Tm (103 °C) aromatic polyester), was greater than those of other AAC. It was assumed that the rigidity of the aromatic ring in the AAC chains influenced their biodegradability with this lipase.

Another synthetic AAC containing adipic acid and terephthalic acid can also be attacked by microorganisms [38]. Kleeberg *et al*. evaluated the biodegradation of AAC synthesized from 1,4-butanediol, adipic acid, and terephtalic acid. *Thermobifida fusca* (known previously as *Thermomonospora fusca*) isolated from compost, showed 20-fold higher degradation rates than usually observed in a common compost test [39]. A thermophilic hydrolase from *Thermobifida fusca* was found to be inducible not only by AAC but also by esters. This enzyme was classified as a serine hydrolase with high similarity to triacylglycerol lipase from *Streptomyces albus* G and triacylglycerol-aclyhydrolase from *Streptomyces* sp. M11 [40].

## 5. Aliphatic Polyesters from Renewable Resources

### 5.1. Poly(3-Hydroxybutyrate) (PHB)

PHB ([-O(CH_3_)CHCH_2_CO-]n) is a natural polymer produced by many bacteria as a means to store carbon and energy. This polymer has attracted research and commercial interest worldwide because it can be synthesized from renewable low-cost feedstocks and the polymerizations are operated under mild process conditions with minimal environmental impact. Furthermore, it can be biodegraded in both aerobic and anaerobic environments, without forming any toxic products. Chowdhury reported for the first time the PHB-degrading microorganisms from *Bacillus*, *Pseudomonas* and *Streptomyces* species[41]. From then on, several aerobic and anaerobic PHB-degrading microorganisms have been isolated from soil (*Pseudomonas lemoigne*, *Comamonas* sp. *Acidovorax faecalis, Aspergillus fumigatus* and *Variovorax paradoxus*), activated and anerobic sludge (*Alcaligenes faecalis, Pseudomonas, Illyobacter delafieldi*), seawater and lakewater (*Comamonas testosterone, Pseudomonas stutzeri*) [42]. The percentage of PHB-degrading microorganisms in the environment was estimated to be 0.5–9.6% of the total colonies [10]. Majority of the PHB-degrading microorganisms were isolated at ambient or mesophilic temperatures and very few of them were capable of degrading PHB at higher temperature. Tokiwa *et al.* emphasized that composting at high temperature is one of the most promising technologies for recycling biodegradable plastics and thermophilic microorganisms that could degrade polymers play an important role in the composting process [43]. Thus, microorganisms that are capable of degrading various kinds of polyesters at high temperatures are of interest. A thermophilic *Streptomyces* sp. isolated from soil can degrade not only PHB but also PES, PBS and poly[oligo(tetramethylene succinate)-co-(tetramethylene carbonate)] (PBS/C). This actinomycete has higher PHB-degrading activity than thermotolerant and thermophilic *Streptomyces* strains from culture collections [44]. A thermotolerant *Aspergillus* sp. was able to degrade 90% of PHB film after five days cultivation at 50 °C [18]. Furthermore, several thermophilic polyester degrading actinomycetes were isolated from different ecosystems. Out of 341 strains, 31 isolates were PHB, PCL and PES degraders and these isolates were identified as members of the genus *Actinomadura, Microbispora*, *Streptomyces*, *Thermoactinomyces* and *Saccharomonospora* [45].

### 5.2. Poly(Lactic Acid) (PLA)

PLA ([-O(CH_3_)CHCO-]n) is a biodegradable and biocompatible thermoplastic which can be produced by fermentation from renewable resources. It can also be synthesized either by condensation polymerization of lactic acid or by ring opening polymerization of lactide in the presence of a catalyst. This polymer exists in the form of three stereoisomers: poly(l-lactide) (l-PLA), poly(d-lactide) (d-PLA) and poly(dl-lactide) (dl-PLA). The manufacture of PLA from lactic acid was pioneered by Carothers in 1932 [46].

Ecological studies on the abundance of PLA-degrading microorganisms in different environments have confirmed that PLA-degraders are not widely distributed, and thus it is less susceptible to microbial attack compared to other microbial and synthetic aliphatic polymers [10,11,34]. The degradation of PLA in soil is slow and that takes a long time for degradation to start [47,48].

Microbial degradation of PLA using *Amycolatopsis* sp. was first reported by Pranamuda *et al.* [11]. Since then, a number of research studies dealing with microbial and enzymatic degradation of PLA have been published [49]. Many strains of genus *Amycolatopsis* and *Saccharotrix* were able to degrade both PLA and silk fibroin. The main amino acid constituents of silk fibroin are l-alanine and glycine and there is a similarity between the stereochemical position of the chiral carbon of l-lactic acid unit of PLA and l-alanine unit in the silk fibroin. Silk fibroin is one of the natural analogues of poly(l-lactide), thus, the PLA degrading microorganisms may probably identify the l-lactate unit as an analogue of l-alanine unit in silk fibroin. Several proteinous materials such as silk fibroin, elastin, gelatin and some peptides and amino acids were found to stimulate the production of enzymes from PLA-degrading microorganisms [50–54].

Williams [55] investigated the enzymatic degradation of PLA using proteinase K, bromelain and pronase. Among these enzymes, proteinase K from *Tritirachium album* was the most effective for PLA degradation. Proteinase K and other serine proteases are capable of degrading l-PLA and dl-PLA but not d-PLA. Furthermore, proteinase K preferentially hydrolyzes the amorphous part of l-PLA and the rate of degradation decreases with an increase in the crystalline part [56,57]. Fukuzaki *et al*. reported that the degradation of PLA oligomers was accelerated by several esterase-type enzymes, especially *Rhizopus delemar* lipase [58]. The purified PLA depolymerase from *Amycolatopsi*s sp. was also capable of degrading casein, silk fibroin, Suc-(Ala)_3_-*p*NA but not PCL, PHB and Suc-(Gly)_3_-*p*NA [50]. Their studies showed that PLA depolymerase was a kind of protease and not a lipase.

It was reported that α-chymotrypsin can degrade PLA and PEA with lower activity on poly(butylene succinate-co-adipate) (PBS/A). Moreover, several serine proteases such as trypsin, elastase, subtilisin were able to hydrolyze l-PLA [59].

## 6. Polymer Blends

### 6.1. Blends of Polyester with Other Polymers

The blending of biodegradable polymers is one approach of reducing the overall cost of the material and modifying the desired properties and degradation rates. Compared to copolymerization method, blending may be a much easier and faster way to achieve the desired properties. More importantly, through blending, other less expensive polymers could be incorporated with one another. Miscibility of the blends is one of the most important factors affecting the final polymer properties. Some of the advantages of producing miscible blends are: single phase morphology and reproducibility of the mechanical properties. However formation of miscible blends especially with non-biodegradable polymers can slow down or even inhibit the degradation of the biodegradable components.

Iwamoto *et al*. developed blend plastics by combining PCL with conventional plastics such as low density polyethylene (LDPE), polypropylene (PP), polystyrene (PS), nylon 6 (NY), poly(ethylene terephthalate) (PET) and PHB, and evaluated their enzymatic degradabilities. The blends of PCL and LDPE, PCL and PP retained the high biodegradability of PCL. In contrast, the degradability of the PCL part in the blends of PCL and PS, PCL and PET, PCL and PHB dropped off remarkably. In case of blends of PCL and NY or PS, the biodegradability of PCL did not change so much. In general, it seems that the higher the miscibility of PCL and conventional plastics, the harder the degradation of PCL on their blends by *R. arrhizus* lipase [60]. Furthermore it was found that degradabilities of PCL/LDPE [61] and PCL/PP [62] blends by the lipase could be controlled, depending on their phase structure.

Different blends of PHB have been performed with biodegradable and non-biodegradable polymers and polysaccharides. The miscibility, morphology and biodegradability of PHB blends with PCL, PBA, and poly(vinyl acetate) (PVAc) were investigated. PHB/PCL and PHB/PBA blends were immiscible in the amorphous state while PHB/PVAc are miscible. Enzymatic degradation of these blends was carried out using PHB depolymerase from *Alcaligenes feacalis* T1. Results showed that the weight loss of the blends decreased linearly with increase in the amount of PBA, PVAc or PCL [63].

Koyama and Doi studied the miscibility, morphology and biodegradability of PHB/PLA blend. The spherulites of the blends decreased with an increase in the content of the PLA and the rate of enzymatic surface erosion also decreased with increasing PLA content in the blend. It was evident that polymer blends containing PHB usually showed improved properties and biodegradability when compared with pure PHB [64].

Different blends of l-PLA/PCL (75/25, 50/50, 25/75) were prepared and enzymatic degradation was observed using proteinase K or *Pseudomonas lipase*. Proteinase K was able to degrade the amorphous domain of PLA but not the crystalline part of l-PLA or PCL. On the contrary, *Pseudomonas* lipase can degrade both the amorphous and crystalline part of PCL, but not l-PLA [65].

### 6.2. Blends of Polyesters and Starch

Blends of synthetic polymers and starch offer cost performance benefits because starch is renewable, cheap and available year-round. In this case the starch blended can be in the form of granules or gelatinized starch or even starch which has been modified chemically to a thermoplastic. It is generally known that blends of PCL and granular starch exhibit a high degree of biodegradation [61].

Takagi *et al*. developed PCL/gelatinized starch blends using corn starch acetates and evaluated their biodegradabilities by an enzyme, α-amylase. Their biodegradabilities rapidly decreased with an increase in PCL content [66].

The feasibility of producing PCL/granular starch blends using different starch from cassava, sago and corn was reported by Pranamuda *et al.* Both tensile strength and elongation of the blends decreased as the starch content increased. The blends were not good in tensile strength but were relatively good in elongation. Continuous phase dispersion of the starch in the PCL was observed in the films using SEM. Degradation of PCL/starch blends showed that as the starch content increased, the polymer blends became more biodegradable using lipase. This could be attributed to the increase in the surface area of PCL after blending with starch, thereby rendering it more susceptible to biodegradation [67].

Noomhorm *et al*. developed PCL/tapioca starch (granular and gelatinized) blends using poly(dioxolane), a poly(ethylene oxide-*alt*-methylene oxide), as compatibilizer. Their biodegradabilities by α-amylase increased as the starch content increased, but were independent of the dispersion of starch in the PCL matrix [68].

PLA and starch are good candidates for polymer blends because both are biodegradable and derivable from renewable resources. Starch can improve the biodegradability and lower the cost while PLA can control the mechanical properties of the blend. However, starch is a hydrophilic material, which does not interact well with hydrophobic polyesters resulting to unfavorable qualities of the blends. In line with this, several approaches have been proposed and developed to overcome the problem of incompatibility of starch and synthetic polymer blends [69,70]. Very good interfacial adhesions of PLA/starch blends were achieved by grafting PLA using maleic anhydride (MA) [71]. Jang *et al*. investigated the interfacial adhesion between PLA and starch using MA and maleated thermoplastic starch (MATPS). Scanning electron microscopy (SEM) showed that MA is a good compatibilizer and PLA/starch blends had increased crystallinity. On the other hand, MATPS is not effective for PLA/starch blends. PLA/starch blends which were compatibilized with MA showed higher biodegradability than ordinary PLA/starch blends at the same PLA ratio [72].

The properties and biodegradability of PBS/A and corn starch (5%–30% w/w) blends were investigated by Ratto *et al*. Results showed that the tensile strength decreased with increase in starch content. Soil burial test showed that the rate of biodegradation increased significantly when the starch content was increased to 20%. It was confirmed that the molecular weight of PBS/A decreased after soil burial indicating that biodegradation was enhanced by the presence of starch [73].

## 7. Polycarbonates

Aliphatic polycarbonates are known to have greater resistance to hydrolysis than aliphatic polyesters. Imai *et al*. first reported the biodegradation of poly(ethylene carbonate) (PEC) implanted in dog tissue and suggested that pronase treatment might be effective in diminishing the PEC mass [74]. Kawaguchi *et al.* reported that PEC was degraded enzymatically in the peritoneal cavity of rats, but not with poly(propylene carbonate) (PPC) [75].

The distribution of PEC (Mn 50,000)-degrading microorganisms seems to be limited, although PPC (Mn 50,000) appears to be non-biodegradable. The percentage of PEC-degrading microorganisms among total colonies ranged from 0.2% to 5.7% [76].

Suyama *et al.* isolated poly(hexamethylene carbonate) (PHC, Mn 2000)-degrading microorganisms which were phylogenetically diverse. *Roseateles depolymerans* 61A formed di(6-hydroxyhexyl) carbonate and adipic acid from PHC, and di(4-hydroxybutyl)-carbonate and succinic acid from poly(butylene carbonate) (PBC, Mn 2,000) [77]. Pranamuda *et al*. found that *Amycolatopsis* sp. HT-6 degraded high molecular-weight poly(butylene carbonate) (PBC, Mn 37,000). In a liquid culture containing 150 mg of PBC film, 83 mg of film was degraded after seven days cultivation [78].

Suyama and Tokiwa reported that a cholesterol esterase from *Candida cylindracea*, lipoprotein lipase from *Pseudomonas* sp., and lipase from *C. cylindracea*, *Chromobacterium viscosus*, porcine pancreas, *Pseudomonas* sp., and *R. arrhizus* degraded PBC (Mn 2000). Lipase and lipoprotein lipase from *Pseudomonas* sp. could also degrade high molecular-weight PBC (Mn 30,000). Lipoprotein lipase from *Pseudomonas* sp produced 1,4-butanediol, CO_2_ and di(4-hydroxybutyl) carbonate from PBC [79].

## 8. Polyurethanes (PU)

PU have various applications such as in the manufacture of plastic foams, cushions, rubber goods, synthetic leathers, adhesives, paints and fibers. There are two types of polyurethanes, that is, the ester type and the ether type. Most commercial polyurethane products are composed of soft segments derived from the polymer-diol, *e.g.,* PCL-diol, polyethylene glycol, poly-tetramethylene glycol, and hard segments from the diisocyanate, *e.g.,* 1,6-hexamethylene-diisocyanate (HDI), diphenylmethane-4,4’-diisocyanate (MDI), tolylene-2,4-diisocyanate (TDI), and diols such as ethylene glycol and butanediol.

Darby and Kaplan reported that polyester-type polyurethanes (ES-PU) were more susceptible to fungal attack than polyether-type polyurethanes (ET-PU) [80]. Tokiwa *et al*. found that *R. delemar* lipase and hog pancreatic lipase can hydrolyze the ES-PU composed of MDI, PCL-diol (Mn 2,000) and 1,4-tetramethylenediol (molar ratio 2:1:1). The amount of degradation products obtained from the ES-PU film with hog pancreatic lipase was approximately half of that produced by *R. delemar* lipase (53% degradation of the original ES-PU film) after 24 h reaction. Hydrolysis rates of ES-PU containing either MDI or TDI were lower than that of ES-PU containing HDI. Thus, it was suggested that the rigidity of ES-PU molecules based on the aromatic rings, rather than the hydrogen bonds among the ES-PU chains, would influence their biodegradability by *R. delemar* lipase [21].

Crabbe *et al.* reported on the degradation of an ES-PU and the secretion of an enzyme-like factor with esterase properties, by *Curvularia senegalensis,* a fungus isolated from soil [81]. Subsequently, Nakajima-Kambe *et al*. showed that *Comamonas acidovorans* strain TB-35 was able to degrade ES-PU made from poly(diethylene adipate) (Mn 2,500 and 2,690) and TDI. A purified ES-PU-degrading enzyme from *C. acidovorans* TB-35, a type of esterase, hydrolyzed the ES-PU and released diethylene glycol and adipic acid [82].

Santerre *et al*. [83] and Wang *et al*. [84] reported that cholesterol esterase from bovine pancreas degraded ES-PU synthesized from TDI, PCL-diol (Mn 1,250) and ethylenediamine, and released the hard-segment components.

However, it seems that no microbe can degrade polyurethane completely, and therefore, it is difficult to clarify the fate of residues after degradation of ES-PU by both microorganisms and enzymes. Furthermore, it is difficult to determine whether ET-PU itself was degraded by microbes to any significant extent.

## 9. Polyamide (Nylon)

### 9.1. Nylon 6

Polyamide (nylon) has excellent mechanical and thermal properties, good chemical resistance and low permeability to gases, but it is known to be resistant to degradation in the natural environment. The poor biodegradability of nylon in comparison with aliphatic polyesters is probably due to its strong interchain interactions caused by the hydrogen bonds between molecular chains of nylon. Some microorganisms such as *Flavobacterium* sp. [85] and *Pseudomonas* sp. (NK87) [86] have been reported to degrade oligomers of nylon 6, but they cannot degrade nylon 6 polymers. Moreover, some white rot fungal strains were reported to degrade nylon 66 through oxidation processes [87].

### 9.2. Nylon 4

It has been reported that nylon 4 was degraded in the soil [88] and in the activated sludge [89]. The results confirmed that Nylon 4 is readily degradable in the environment. Furthermore, the biodegradability of nylon 4 and nylon 6 blends was investigated in compost and activated sludge. The nylon 4 in the blend was completely degraded in 4 months while nylon 6 was not degraded [90]. Recently, Yamano *et al*. was able to isolate polyamide 4 degrading microorganisms (ND-10 and ND-11) from activated sludge. The strains were identified as *Pseudomonas* sp. The supernatant from the culture broth of strain ND-11 degraded completely the emulsified nylon 4 in 24 h and produced γ-aminobutyric acid (GABA) as degradation product [91].

Generally speaking, degradation of polyamides is still unclear. Thus further investigations on the pathways of degradation are necessary.

### 9.3. Copolyamide-Esters (CPAE)

In order to improve the properties of biodegradable aliphatic polyesters for various fields of applications and to find the reason why industrialized aliphatic polyamides (nylon) are not biodegradable, CPAE were synthesized by the amide-ester interchange reaction between PCL and various nylons. The susceptibility of CPEA to hydrolysis by *R. delemar* lipase decreased with shortening of the nylon blocks in CPAE chains and with increasing nylon content. The simple blends of PCL and nylon retained high biodegradability of PCL. Thus it was assumed that the amount and distribution of hydrogen bonds, based on the amide bonds, in the CPAE chains influenced their biodegradability by this lipase [92].

Komatsu *et al*. synthesized CPAE from ɛ-caprolactam with ɛ-caprolactone or δ-valerolactone by ring-opening copolymerization using Na catalyst under reduced pressure, and examined their degradation by *R. arrhizus* lipase. Most CPAEs were degraded by the lipase. The biodegradability decreased with an increase in Tm of CPAEs as a result of an increase in the amide bond contents [93].

It would be very important that various types of interaction among macromolecular chains, which are related to Tm and storage modulus, are taken into consideration when designing the biodegradable solid polymers.

## 10. Polyethylene (PE)

PE is a stable polymer, and consists of long chains of ethylene monomers. PE cannot be easily degraded with microorganisms. However, it was reported that lower molecular weight PE oligomers (MW = 600–800) was partially degraded by *Acinetobacter* sp. 351 upon dispersion, while high molecular weight PE could not be degraded [94].

Furthermore, the biodegradability of low density PE/starch blends was enhanced with compatibilizer [95]. Biodegradability of PE can also be improved by blending it with biodegradable additives, photo-initiators or copolymerization [96,97]. The initial concept of blending PE with starch was established in UK to produce paper-like PE bag. A few years later, the idea to blend PE with starch and photoinitiators was conceived in the US as a way of saving petroleum, though its biodegradability was also taken into account.

Environmental degradation of PE proceeds by synergistic action of photo-and thermo-oxidative degradation and biological activity (*i.e.,* microorganisms). When PE is subjected to thermo- and photo-oxidization, various products such as alkanes, alkenes, ketones, aldehydes, alcohols, carboxylic acid, keto-acids, dicarboxylic acids, lactones and esters are released. Blending of PE with additives generally enhances auto-oxidation, reduces the molecular weight of the polymer and then makes it easier for microorganisms to degrade the low molecular weight materials. It is worthy to note that despite all these attempts to enhance the biodegradation of PE blends, the biodegradability with microorganisms on the PE part of the blends is still very low.

## 11. Polypropylene (PP)

PP is a thermoplastic which is commonly used for plastic moldings, stationary folders, packaging materials, plastic tubs, non-absorbable sutures, diapers etc. PP can be degraded when it is exposed to ultraviolet radiation from sunlight. Furthermore, at high temperatures, PP is oxidized. The possibility of degrading PP with microorganisms has been investigated [98].

## 12. Polystyrene (PS)

PS is a synthetic hydrophobic polymer with high molecular weight. PS is recyclable but not biodegradable. Although it was reported that PS film was biodegraded with an *Actinomycete* strain, the degree of biodegradation was very low [99]. At room temperature, PS exists in solid state. When it is heated above its glass transition temperature, it flows and then turns back to solid upon cooling. PS being a transparent hard plastics is commonly used as disposable cutleries, cups, plastic models, packing and insulation materials.

## 13. Conclusions and Future Prospects

Biodegradable plastic is an innovative means of solving the plastic disposal problem from the standpoint of development of new materials. In general, plastics are water-insoluble, thermo-elastic polymeric materials. Biodegradability of plastics is affected by both their chemical and physical properties. Beside the covalent forces of polymer molecules, various kinds of weak forces (*i.e.,* hydrogen bond forces, van der Waals forces, coulombic forces, etc.) among macromolecular chains affect not only the formation of polymer aggregates, but also the structure and physical properties and function (reactivity) of the polymer aggregates. The biodegradation mechanisms of plastics as shown in this review can be applied to biomass that are composed of polymeric materials (*i.e.,* cellulose, hemicellulose, lignin, chitin, silk fibroin, etc.).

Furthermore, knowledge about the biodegradation mechanisms of plastics would be useful for studies on protein conformational diseases that are associated with aggregation, deposition and crystallization of abnormal proteins such as Alzheimer’s disease and bovine spongiform encephalopathy (BSE). Proteinase K and l-PLA-degrading enzyme from *Amycolatopsis* sp. can degrade both l-PLA and silk fibroin. It is well known that proteinase K can degrade prion protein, of which mis-folded form of it is resistant to proteinase K and is implicated in BSE in cattle. Polyesters (*i.e.,* l-PLA, d-PLA, PCL, PHB) can be used as a model for abnormal protein, because it is easy to change their high order structures by quenching and elongation, etc. and to evaluate their rate of enzymatic degradation.

Lipolytic enzymes such as lipase and esterase can hydrolyze not only fatty acid esters and triglycerides, but also aliphatic polyesters. We can understand that lipolytic enzyme has an important role in the degradation of natural aliphatic polyesters such as cutin, suberin and esteroid in the natural environment and animal digestive tract. However, it is not certain whether human body produces any aliphatic polyesters or not.

## Figures and Tables

**Table 1.** Chemical structures of aliphatic polyester, polycarbonate, polyurethanes and polyamides with their (Tm)s.

**Name**	**Chemical Structure**	**Tm (°C)**
Polyester	-O-(CH_2_)_6_-O-CO-(CH_2_)_4_-CO-	60
Polycarbonate	-O-(CH_2_)_4_-O-CO-O-(CH_2_)_4_-O-CO-	65
Polyurethane	-NH-(CH_2_)_6_-NH-CO-O-(CH_2_)_4_-O-CO-	180
Polyamide	-NH-(CH_2_)_6_-NH-CO-(CH_2_)_6_-CO-	240
Polyamide	-NH-(CH_2_)_6_-NH-CO-(CH_2_)_4_-CO-	265
